# Case Report: Non-Catheter-Related arterial hemorrhage as a complication of hemoperfusion in hypertriglyceridemic pancreatitis: mechanistic hypotheses and multidisciplinary strategies

**DOI:** 10.3389/fmed.2025.1670782

**Published:** 2025-10-02

**Authors:** Pochen Li, Yang Wu, Danxia Ge, Ruyi Xu, Qianping Zhang, Yujiao Li, Lingyao Zhang, Fangyu Yu

**Affiliations:** ^1^Intensive Care Unit, Ningbo Municipal Hospital of Traditional Chinese Medicine (TCM), Affiliated Hospital of Zhejiang Chinese Medical University, Ningbo, China; ^2^Department of Respiratory, Ningbo Municipal Hospital of Traditional Chinese Medicine (TCM), Affiliated Hospital of Zhejiang Chinese Medical University, Ningbo, China

**Keywords:** hypertriglyceridemia-induced acute pancreatitis, hemoperfusion, spontaneous hemorrhage, anticoagulation strategy, endovascular intervention

## Abstract

Hypertriglyceridemia-induced acute pancreatitis (HTG-AP) is a rapidly progressive and increasingly prevalent subtype of acute pancreatitis. Hemoperfusion (HP) is commonly employed as a prompt and effective method to lower serum triglyceride (TG) levels. However, in the context of anticoagulant administration and underlying coagulopathy, this approach may precipitate severe hemorrhagic complications. We report a case involving a female patient with HTG-AP who underwent HP for markedly elevated TG levels. Upon admission, the patient exhibited mildly prolonged thrombin time. Following the second session of HP, she developed hemorrhagic shock. Imaging revealed massive hemoperitoneum initially suspected to result from venous catheterization. Subsequent digital subtraction angiography (DSA) confirmed active arterial bleeding from a branch of the right internal iliac artery, which was successfully managed by embolization. Post-procedural evaluation suggested that the arterial rupture was likely due to increased vascular fragility caused by systemic inflammation from acute pancreatitis, further aggravated by anticoagulant exposure during HP. This case underscores the critical importance of pre-treatment bleeding risk assessment, especially in patients with pre-existing coagulation abnormalities. In cases of acute hemorrhage, clinicians must remain alert to non-iatrogenic bleeding sources associated with the underlying pathology and therapeutic interventions. Individualized anticoagulation strategies and vigilant hemodynamic and coagulation monitoring are essential to mitigate the risk of treatment-associated hemorrhagic events.

## 1 Introduction

Hypertriglyceridemia-induced acute pancreatitis (HTG-AP) is a distinct subtype of acute pancreatitis, increasingly observed in specific populations (1). It is characterized by rapid progression and a high risk of multi-organ dysfunction, posing significant clinical challenges. Studies have demonstrated that when serum triglyceride (TG) levels exceed 11.3 mmol/L, intensified pancreatic lipolysis leads to excessive free fatty acid (FFA) release (2). These FFAs exert direct cytotoxic effects on pancreatic parenchyma and capillary endothelium, triggering local injury and systemic inflammatory response syndrome (SIRS). To rapidly clear circulating lipids and arrest disease progression, blood purification techniques–particularly hemoperfusion (HP) –have been widely adopted (3, 4).

While extracorporeal blood purification offers effective early intervention, it introduces a non-negligible risk of bleeding, primarily due to anticoagulation (5). Furthermore, acute pancreatitis itself may predispose patients to spontaneous hemorrhage. Recent reports have highlighted cases of deep arterial bleeding during HP unrelated to vascular access injury (6, 7).

We present a rare case of massive abdominopelvic hemorrhage in a patient with HTG-AP undergoing routine HP. The bleeding originated from a branch of the internal iliac artery remote from the catheterization site. Through this case, we aim to enhance clinical awareness of spontaneous arterial bleeding associated with HP, especially in the context of underlying coagulopathy.

## 2 Case presentation

A 30-year-old woman was admitted via the emergency department on April 2, 2025, with abdominal pain lasting 10 h. On presentation, her temperature was 37.3 °C; blood pressure, 130/97 mmHg; heart rate, 66 bpm; and respiratory rate, 14 breaths/min. Physical examination revealed clear consciousness, normal skin and mucosa, and marked tenderness in the left upper abdominal quadrant. The patient denied any history of bleeding disorders, anticoagulant or antiplatelet use, and was not menstruating at presentation.

Initial laboratory tests revealed a markedly elevated TG level of 35.91 mmol/L, serum amylase at 204 U/L, and urinary amylase at 1016 U/L. Non-contrast abdominal CT demonstrated pancreatic tail swelling with hypodense areas and mild peripancreatic exudation, consistent with HTG-AP. Coagulation studies showed prolonged thrombin time (TT) at 29.1 s and mildly elevated fibrinogen. Additional labs included Alanine Aminotransferase (ALT) 19 U/L, Aspartate Aminotransferase (AST) 18 U/L, creatinine 35 μmol/L, Blood Urea Nitrogen (BUN) 3.47 mmol/L, potassium 4.06 mmol/L, sodium 130.6 mmol/L, and total calcium 2.22 mmol/L. Blood routine showed White Blood Cell count (WBC) 16.5 × 10^9^/L, Red Blood Cell count (RBC) 4.67 × 10^12^/L, Platelet count (PLT) 228 × 10^9^/L, Hemoglobin (HGB) 163 g/L, and C-Reactive Protein (CRP) 6.2 mg/L.

The patient received intravenous esomeprazole 40 mg q12h, somatostatin 3 mg q12h, and phloroglucinol 100 mg BID, along with fluid resuscitation, insulin, and 10% glucose. HP was initiated to lower TG levels. A right femoral venous catheter was placed under ultrasound guidance using a standardized protocol, with correct positioning confirmed. HP was conducted using the MG330 cartridge, with each session lasting 2 h. A total of 3 sessions were scheduled. Anticoagulation consisted of sodium heparin with a loading dose of 3000 U followed by continuous infusion at 1500 U/h.

**FIGURE 1:**
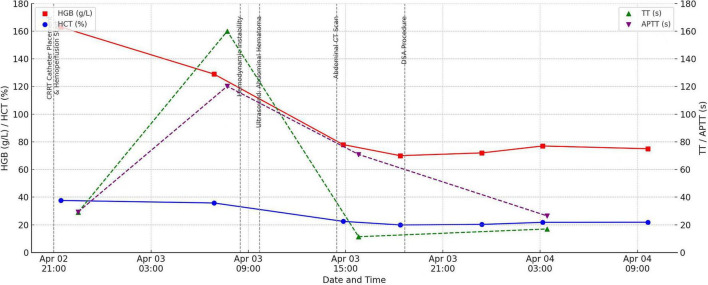
Trends in Hemoglobin (HGB), Hematocrit (HCT), Activated Partial Thromboplastin Time (APTT), and Thrombin Time (TT), annotated with key clinical events.

Approximately 6 h after the second session, the patient developed sudden tachycardia (>120 bpm), hypotension (nadir SBP 80 mmHg), and abdominal distension. Physical examination revealed positive shifting dullness. HGB dropped acutely from 129 g/L to 78 g/L–a 51 g/L decline–suggesting concealed intra-abdominal hemorrhage. Hematocrit also declined significantly. β-hCG was negative (Figure 1).

**FIGURE 2:**
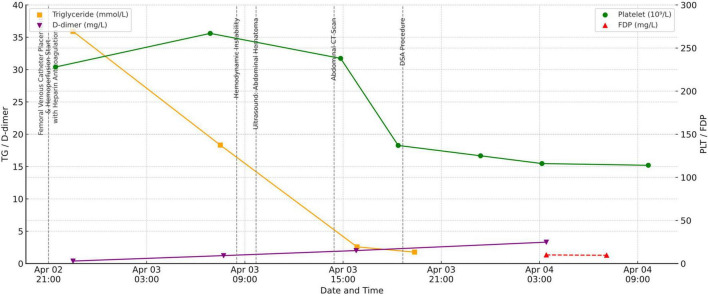
Trends in platelet count (PLT), D-dimer, fibrin degradation products (FDP), and triglycerides (TG), annotated with key events.

During this period, dynamic laboratory monitoring demonstrated a progressive decline in PLT counts (228 × 10^9^/L at admission, 267 × 10^9^/L on April 3 at 06:53, 238 × 10^9^/L at 14:51, 137 × 10^9^/L at 18:22, and 114 × 10^9^/L on April 4 at 09:39). Meanwhile, D-dimer increased from 0.39 mg/L at admission to 1.23 mg/L, 2.01 mg/L, and 3.31 mg/L, while fibrin degradation products (FDP) rose to 10.0 and 9.6 mg/L during the acute hemorrhagic phase. In parallel, TG decreased markedly following HP: from 35.91 mmol/L at admission to 18.32 mmol/L after the first treatment, and further down to 2.58 mmol/L and 1.77 mmol/L after subsequent sessions (Figure 2).

**FIGURE 3:**
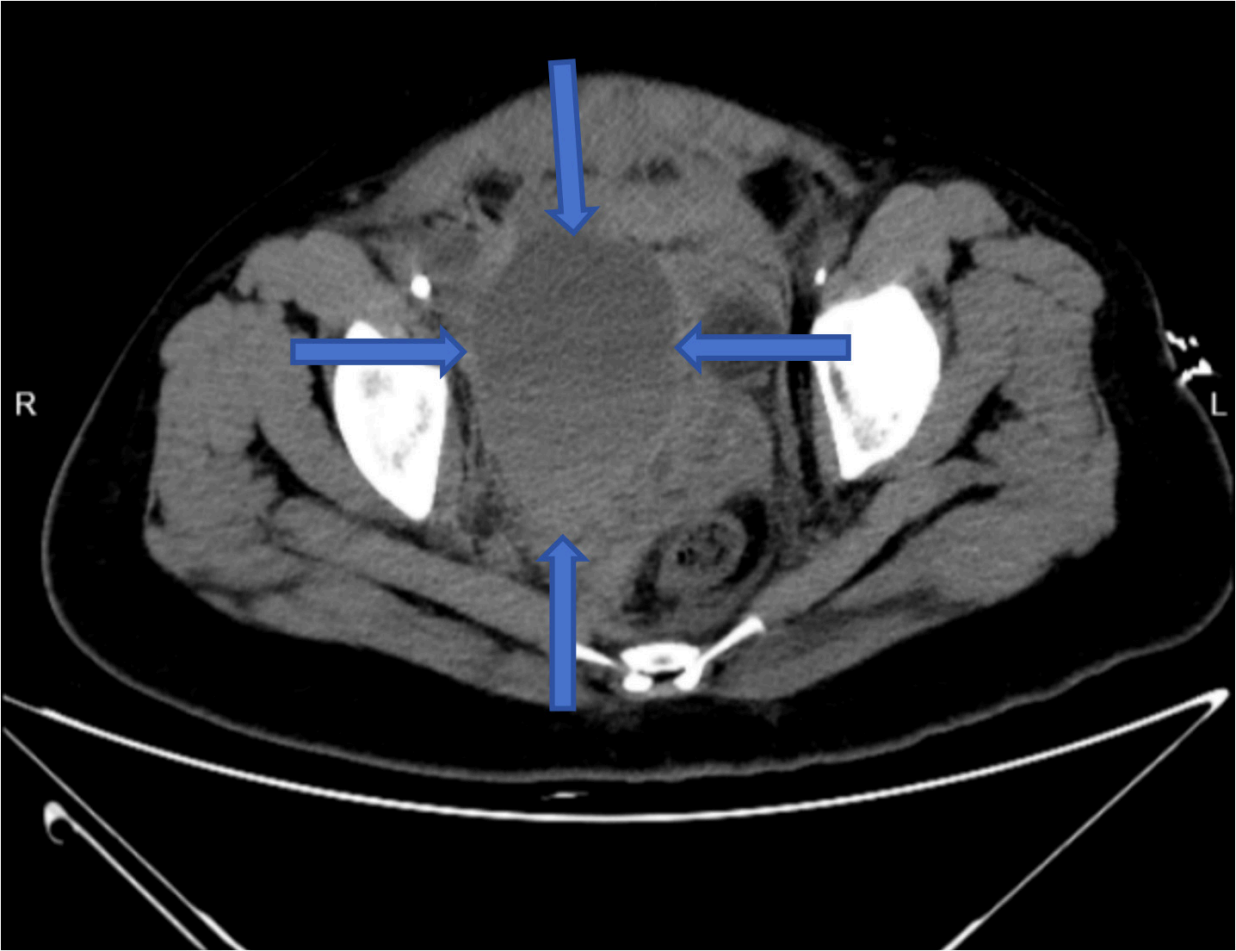
Axial abdominal CT scan showing a hematoma (indicated by arrow).

Resuscitation included fluid replacement, transfusion of leukocyte-depleted red cells and fresh frozen plasma, and norepinephrine infusion. Bedside ultrasound showed intra-abdominal fluid and a hypoechoic pelvic mass. Abdominal CT confirmed hemoperitoneum with loculated fluid collections up to 106 × 77 mm. Emergency digital subtraction angiography (DSA) was performed (Figure 3).

**FIGURE 4:**
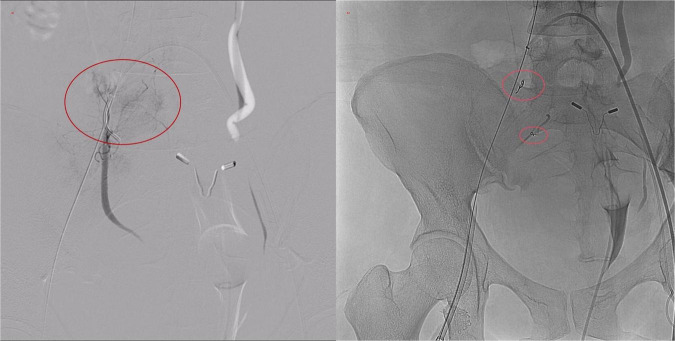
Digital subtraction angiography (DSA) images. **(a)** Active contrast extravasation from a distal branch of the internal iliac artery. **(b)** Post-embolization angiogram demonstrating successful occlusion of the bleeding site with metallic coils.

DSA revealed no extravasation along the venous access or iliac vein trajectory. The right femoral, common iliac, and external iliac arteries appeared intact. However, active contrast extravasation was identified in a branch of the right internal iliac artery. Endovascular embolization was performed, yielding immediate hemodynamic improvement (Figure 4). Post-procedure HGB levels stabilized between 72–77 g/L at 1, 4, and 10 h postoperatively, and the patient’s condition gradually improved.

Following embolization and supportive care, the patient’s condition stabilized and she was successfully transferred out of the ICU. She was discharged on April 26. At outpatient follow-up on May 13, her clinical status remained stable, with HGB 117 g/L, RBC count 4.41 × 10^12^/L, TG 2.39 mmol/L, and serum amylase 48 U/L. No recurrent hemorrhage or relapse of pancreatitis was observed during the follow-up period.

## 3 Discussion

Acute pancreatitis (AP) is a common abdominal emergency that may lead to local necrosis, abscess formation, and systemic complications (8). Although HTG-AP often has a favorable prognosis when promptly treated, spontaneous hemorrhagic events–including intra-abdominal and retroperitoneal bleeding–may occur in severe cases, posing considerable risk (9). Literature reports indicate that potential hemorrhagic vascular complications are associated with a mortality rate of 34%–52% in cases of AP (6). Arterial bleeding, though rare, is more abrupt and life-threatening. For example, in 2023, Yamazaki M et al. (10) described a case of acute pancreatitis complicated by rupture of a gastroduodenal artery pseudoaneurysm, which was successfully treated via surgical intervention. A 2022 case report in Frontiers in Surgery documented a retroperitoneal hemorrhage secondary to pancreatitis-associated pancreatic pseudocyst rupture (11).

In this case, hemorrhagic shock occurred after the second HP. DSA confirmed that the hemorrhage originated from a remote internal iliac artery branch, unrelated to catheterization, prompting further investigation into the underlying mechanism. Based on interdisciplinary discussion, we propose a multifactorial mechanism:

(1)    Disease-related vascular fragility: In HTG-AP, pancreatic lipase–mediated hydrolysis of triglycerides leads to the release of FFAs, which exert direct cytotoxic effects on both pancreatic acinar and vascular endothelial cells. This FFA-mediated injury induces acinar necrosis and disrupts endothelial barrier integrity through oxidative stress, nitric oxide inhibition, and activation of TLR4/NF-κB–dependent inflammatory pathways. The resultant inflammatory cascade, characterized by neutrophil infiltration and cytokine release, increases microvascular permeability and compromises vessel wall stability. Consequently, this cascade may predispose patients to spontaneous hemorrhage under physiological stress, even in the absence of visible pseudoaneurysms (12, 13).
(2)    Hemoperfusion-induced coagulation disturbances: Adsorption cartridges may non-selectively remove coagulation proteins such as fibrinogen, prothrombin, and antithrombin III (14). This patient’s prolonged TT upon admission suggested a baseline coagulation deficit. Combined with systemic anticoagulation, this likely exacerbated bleeding risk, particularly in patients with abnormal APTT or TT.
(3)    Exclusion of autoimmune coagulopathy (15): Post-stabilization, autoimmune screening (ANA, anti-dsDNA, ACL, anti-β2GPI, anti-Sm, anti-SSA, LAC) was negative. Although hereditary or acquired coagulopathy could not be fully excluded due to patient’s financial constraints, no specific factor deficiency was documented.

Although angiography excluded catheter-related vascular injury and demonstrated focal bleeding from a distal branch of the internal iliac artery, the precise etiology of this hemorrhage could not be determined with certainty. While disease-related vascular fragility and systemic anticoagulation were considered the most likely contributors, we acknowledge that iatrogenic factors cannot be entirely excluded. This limitation underscores the importance of maintaining vigilance for both spontaneous and procedure-related causes of bleeding when managing patients with HTG-AP undergoing HP.

It is noteworthy that in patients with acute pancreatitis complicated by hemorrhage, the possibility of disseminated intravascular coagulation (DIC) must always be considered. In this case, dynamic laboratory monitoring showed a progressive decline in platelet counts, together with rising D-dimer levels and elevated FDP, findings consistent with consumptive coagulopathy and raising concern for DIC. However, the absence of systemic microthrombi, the lack of overt ecchymoses or generalized bleeding tendency, and the focal arterial rupture demonstrated by angiography collectively argued against overt DIC. The patient’s stabilization and favorable recovery following targeted embolization further supported this interpretation.

Furthermore, the necessity of HP in HTG-AP remains controversial. Although HP achieves rapid TG reduction, recent evidence has questioned its impact on long-term prognosis, with some studies suggesting no significant improvement in outcomes and a potential association with increased ICU utilization (16). In this case, HP was selected to achieve prompt biochemical control; however, whether HP should be routinely performed in HTG-AP requires further validation through high-quality clinical studies. Therefore, the decision to initiate HP should be made cautiously, balancing the biochemical benefit against the uncertain effect on patient-centered outcomes and the potential risk of additional harm.

Collectively, these mechanisms highlight a multifactorial hemorrhagic risk profile involving disease pathology, procedural anticoagulation, and potential undiagnosed coagulopathy.

## 4 Clinical Implications

This case illustrates that although rare, spontaneous arterial hemorrhage during HP in HTG-AP can be catastrophic. Vigilant risk assessment and timely intervention are essential. In conjunction with the relevant consensus (17), we propose the following recommendations:

(1)    Pre-treatment assessment: Evaluate TT, APTT, FIB, D-dimer, liver and renal function, and–where feasible–platelet function and coagulation factor levels. High-risk patients may benefit from alternative anticoagulation (e.g., regional citrate, heparin-free regimens, or low-dose protocols).

(2)    Enhanced intra- and post-procedural monitoring: Monitor vital signs, urine output, and HGB dynamically during and 6–12 h post-treatment. Early signs (distension, dullness, hypotension) warrant prompt imaging. Do not rely solely on access site inspection–deep arterial sources must be considered.
(3)    Early imaging and interventional strategy: Rapid access to ultrasound, CT, and DSA is critical. Interventional radiology should be integrated into the acute care algorithm for suspected internal hemorrhage.
(4)    Patient communication and multidisciplinary care: Informed consent must include discussion of non-access-related hemorrhage. When bleeding occurs, management by a multidisciplinary team–including intensivists, interventional radiologists, and hepatobiliary surgeons–is essential.
(5)    Future research: Large-scale, prospective studies are needed to identify predictors of HP-associated bleeding and optimize anticoagulation protocols. Innovations in cartridge design may further enhance safety.

## Data Availability

The original contributions presented in this study are included in this article/supplementary material, further inquiries can be directed to the corresponding author.
